# Associations between early childhood appetitive traits and dietary diversity in school-age children: a two-year longitudinal follow-up study

**DOI:** 10.3389/fnut.2026.1824771

**Published:** 2026-07-21

**Authors:** Yuhong Mu, Duhan Bu, Xiaowen Xu, Jing Yuan, Xinxin Dai, Weiqing Lv, Yan Liu, Jiachen Tang, Libing Zhang, Yu Jin

**Affiliations:** 1 Department of Pediatrics, Yangzhou Maternal and Child Health Care Hospital Affiliated to Yangzhou University, Yangzhou, China; 2 Department of Endocrinology, Children's Hospital of Soochow University, Suzhou, China

**Keywords:** appetitive traits, child eating behavior questionnaire, children, dietary diversity, dietary diversity score

## Abstract

**Background:**

Dietary diversity reflects diet quality and is critical for children’s growth and long-term health. Appetitive traits affect dietary habits and long-term food choices. However, few studies have explored the associations between early childhood appetitive traits and dietary diversity in school-age children using standardized and validated tools.

**Methods:**

We recruited 208 kindergarten children aged 5–6 years, with 194 completing the two-year longitudinal follow-up. Appetitive traits were evaluated at age 5–6 using the Child Eating Behavior Questionnaire (CEBQ). Dietary diversity was assessed 2 years later via the Dietary Diversity Score (DDS). Binary logistic regressions were carried out to assess the associations between early childhood appetitive traits and dietary diversity. Inverse probability weighting (IPW) was conducted in complete cases to test the robustness of the main results.

**Results:**

Higher food fussiness at 5–6 years was significantly associated with a lower likelihood of high DDS 2 years later (OR = 0.521, 95% CI: 0.327–0.830). No significant sex interactions were observed for these associations.

**Conclusion:**

Food fussiness at 5–6 years was associated with dietary diversity in school-age children. These findings underscore the relevance of early appetitive traits to dietary diversity and provide evidence-based insights for promoting children’s nutrition.

## 1 Introduction

Nutrition represents a fundamental pillar of human wellbeing across the lifespan, playing a pivotal role in survival and socioeconomic productivity (1). Characterized by profound physical growth and development, childhood needs an especially abundant supply of diverse nutrients for the increased energy requirements, and this crucial stage is sensitive and vulnerable to the detrimental effects of a diet lacking in diversity (2). Not only brain development, growth trajectory and survival rate, but also lifelong health can be affected by nutrient insufficiency in childhood (3). Hence, we should focus on childhood diet diversity, which is modifiable, to avert the long-term adverse consequences of malnourishment, which is irreversible.

Dietary diversity score (DDS), a key indicator evaluating diet quality (4), reflects overall nutritional adequacy based on nine food categories, and has been validated and implemented widely in many studies (5). Existing evidence indicates that inadequate dietary diversity is not uncommon in children (6). Low DDS is associated with multiple adverse health consequences, including obesity (7), dyslipidemia (8), and cognitive impairment (9). Given the far-reaching impacts of these diseases, focus should be put on prevention during childhood.

Appetitive traits can be observed and influence dietary habits and long-term food choices (10). Many studies found that appetitive traits were associated with diet-related diseases, for example obesity (11). Fortunately, children’s appetitive traits could be modified through improving nutritional knowledge (12) and feeding practices of parents (13). Therefore, fostering healthier appetitive traits for children may be a feasible approach to achieve a high-quality diet. The Child Eating Behavior Questionnaire (CEBQ) is the most widely used measure assessing children’s appetitive traits (14). However, to date, few studies have investigated the associations between early childhood appetitive traits and dietary diversity in school-age children, using CEBQ and DDS (4, 14).

This study explored the associations between appetitive traits in children aged 5–6 years attending kindergarten and dietary diversity 2 years later when they were in primary school. We hypothesized that: (1) high food approach traits in 5–6-year-old children attending kindergarten would be associated with a high DDS 2 years later; and (2) high food avoidance traits would be associated with a low DDS 2 years later.

## 2 Materials and methods

### 2.1 Study population

We recruited 208 children from the pediatric outpatient department of Yangzhou Maternal and Child Health Care Hospital between October 2023 and December 2023. All participants went to the hospital for routine physical examinations or minor illnesses that were not expected to affect eating behavior or nutritional status. Inclusion criteria were as follows: (1) being 5–6 years old and attending kindergarten; (2) no history of food allergy; (3) no serious underlying diseases. After inclusion, appetitive traits were assessed, and dietary diversity was evaluated 2 years later. In the end, 194 children finished the follow-up, yielding a follow-up rate of 93.27%. Ethical approval was obtained from the Medical Ethics Committee of Yangzhou Maternal and Child Health Care Hospital Affiliated to Yangzhou University (Ethics number: 2023094). All the participants and their parents participated voluntarily. Verbal assent was obtained from the children and written informed consent was signed by their parents.

### 2.2 Appetitive traits and dietary diversity assessment

Children’s appetitive traits were assessed at age 5–6 years via the Chinese version of the CEBQ completed by their parents. The Chinese version of the CEBQ has been validated among Chinese preschool children (15). It consists of 35 items classifying appetitive traits into eight dimensions (four food approach and four food avoidance traits) (14). Higher scores on food approach subscales indicate a stronger appetite: food responsiveness, enjoyment of food, desire to drink, and emotional overeating. Higher scores on food avoidance subscales indicate a more diminished appetite: satiety responsiveness, slowness in eating, food fussiness, and emotional undereating (14).

DDS was developed based on the guidelines provided by the Food and Agriculture Organization of the United Nations (16). A total of nine food categories were included: starchy staples (such as cereals, white roots, and tubers); dark green leafy vegetables (such as spinach, kale, and broccoli); other fruits and vegetables rich in vitamin A (such as pumpkin, spinach, and mango); other fruits and vegetables (such as cucumber, tomato, and mushroom); organ meat (such as liver, heart, and intestines); meat and fish (such as pork, beef, and salmon); eggs (such as chicken eggs, duck eggs, and quail eggs); legumes, nuts, and seeds (such as chickpea, walnut, and sunflower seed); and milk and milk products (such as fresh milk, yogurt, and cheese). Other food groups, including sugary snacks, flavored beverages, processed sauces, prepared meals, and other manufactured food products, were excluded from the current study. Parents were required to report all food consumed by their children based on 24-h dietary recall. Each food group was scored 1 if consumed during the preceding 24 h, and 0 if not consumed. In this study, low DDS was defined as a score < 5, and high DDS as a score ≥ 5 (17).

### 2.3 Covariates

Trained research staff measured children’s height and weight, and derived the BMI standard deviation score (BMI SD score). Parents reported children’s date of birth, sex, gestational age, birthweight, parental educational level, maternal educational level, and annual household income. Parental and maternal educational levels were classified into high (college or above), medium (vocational training), and low (high school or below). Annual household income was divided by the threshold of 150,000 yuan.

### 2.4 Statistical analysis

Continuous data were expressed as mean ± standard deviation and compared using independent-samples t-tests. Categorical data were reported as frequencies and percentages, and group comparisons were performed with the Chi-square test. Binary logistic regression analysis was conducted to investigate the associations between appetitive traits and dietary diversity. The fully adjusted model adjusted for age at assessing appetitive traits, biological sex, gestational age, birthweight, paternal educational level, maternal educational level, household income, and BMI SD score at 5–6 years. Little’s MCAR test showed that missing data on DDS were missing completely at random (MCAR) (*p* > 0.05). Inverse probability weighting (IPW) was implemented in Stata 16. The core principle of IPW is to assign a weight to each participant who completed the follow-up, so that the weighted samples could better represent the original study population and compensate for the information loss caused by lost-to-follow-up participants (18). Weights were derived from demographic characteristics: age at assessing appetitive traits, sex, gestational age, birthweight, paternal educational level, maternal educational level, household income and BMI SD score at 5–6 years. Significant results were indicated by *p* < 0.05.

## 3 Results

### 3.1 Characteristics of participants

The characteristics of the final study sample compared with those lost to follow-up for DDS 2 years later are shown in Supplementary Table 1. No differences were found between groups in age at assessing appetitive traits, sex, gestational age, birthweight, BMI SD score at 5–6 years, paternal educational level, maternal educational level, or household income.

Table 1 displays the characteristics of the final study sample in low and high DDS groups. No significant differences were found between groups in age at assessing appetitive traits, sex, gestational age, BMI SD score at 5–6 years, or household income. However, participants in the high DDS group had significantly lower birthweight, higher levels of paternal and maternal education (all *p* < 0.05).

**Table 1:** Characteristics of the final study sample in low and high DDS groups.

Characteristics	DDS < 5 (*n* = 85) *n* (%) or mean ± SD	DDS ≥ 5 (*n* = 109) *n* (%) or mean ± SD	*p*-value^1^
Age at assessing appetitive traits, years	5.59 ± 0.32	5.60 ± 0.35	0.845
Sex			0.675
Female	45 (52.94%)	61 (55.96%)	
Male	40 (47.06%)	48 (44.04%)	
Gestational age, weeks	38.95 ± 1.45	39.14 ± 1.85	0.450
Birthweight, g	3473.00 ± 635.47	3310.00 ± 503.81	**0.048**
Sex-adjusted and age-adjusted BMI SD score at 5–6 years	−0.05 ± 1.36	0.04 ± 1.64	0.662
Paternal educational level^2^			**0.001**
High	48 (56.47%)	77 (70.64%)	
Medium	28 (32.94%)	32 (29.36%)	
Low	9 (10.59%)	0 (0.00%)	
Maternal educational level^3^			**0.038**
High	48 (56.47%)	77 (70.64%)	
Medium	32 (37.65%)	31 (28.44%)	
Low	5 (5.88%)	1 (0.92%)	
Household income^4^			0.290
<150,000 yuan	29 (34.12%)	32 (29.36%)	
≥150,000 yuan	56 (65.88%)	77 (70.64%)	

^1^*p*-values reflect differences by characteristic between the low (DDS < 5) and high (DDS ≥ 5) DDS groups and were obtained by *t*-tests or chi-square tests.

^2^Paternal educational level was measured when assessing children’s appetitive traits (high: college or above, medium: vocational training, low: high school or below).

^3^Maternal educational level was measured when assessing children’s appetitive traits (high: college or above, medium: vocational training, low: high school or below).

^4^Household income was measured when assessing children’s appetitive traits.

DDS, dietary diversity score. BMI SD, body mass index standard deviation.

*p*-values < 0.05 are bolded.

### 3.2 Appetitive traits of participants

Supplementary Table 2 compares the appetitive traits between the final study sample and those lost to follow-up for DDS 2 years later. No differences were observed between groups in food responsiveness, enjoyment of food, satiety responsiveness, slowness in eating, or food fussiness. However, participants lost to follow-up exhibited significantly lower scores on desire to drink, emotional overeating, and emotional undereating (all *p* < 0.05).

Table 2 displays appetitive traits in low and high DDS groups. No significant differences were found between groups in food responsiveness, emotional overeating, or emotional undereating. However, participants in the high DDS group had higher scores on enjoyment of food, but significantly lower scores on desire to drink, satiety responsiveness, slowness in eating, and food fussiness (all *p* < 0.05).

**Table 2:** Appetitive traits of the final study sample in low and high DDS groups^1^.

Appetitive traits	DDS < 5 (*n* = 85) Mean ± SD	DDS ≥ 5 (*n* = 109) Mean ± SD	*p*-value^2^
Food approach traits			
Food responsiveness	2.31 ± 0.92	2.18 ± 0.92	0.328
Enjoyment of food	3.13 ± 0.99	3.46 ± 0.89	**0.015**
Desire to drink	2.42 ± 0.99	2.11 ± 0.97	**0.030**
Emotional overeating	2.02 ± 0.74	1.90 ± 0.75	0.266
Food avoidance traits			
Satiety responsiveness	2.84 ± 0.77	2.48 ± 0.75	**0.001**
Slowness in eating	2.99 ± 0.89	2.72 ± 0.96	**0.048**
Food fussiness	3.02 ± 0.87	2.66 ± 0.74	**0.002**
Emotional undereating	2.66 ± 0.60	2.50 ± 0.52	0.064

^1^Measured with the Child Eating Behavior Questionnaire.

^2^*p*-values reflect differences by appetitive traits between the low (DDS < 5) and high (DDS ≥ 5) DDS groups and were obtained by *t*-tests.

DDS, dietary diversity score.

*p*-values < 0.05 are bolded.

### 3.3 Associations between appetitive traits and dietary diversity

The fully adjusted model showed that greater food fussiness at 5–6 years decreased the odds of having a high DDS 2 years later (OR = 0.521, 95% CI: 0.327–0.830). There were no associations between other appetitive traits and DDS (Table 3).

**Table 3:** Binary logistic regression models showing the associations between appetitive traits at 5–6 years and dietary diversity scores at 7–8 years in the final study sample.

Appetitive traits^1^	Model^2^		
	OR	95% CI	*p*-value
Food approach traits			
Food responsiveness	0.852	(0.543, 1.334)	0.483
Enjoyment of food	1.042	(0.643, 1.690)	0.867
Desire to drink	0.771	(0.517, 1.150)	0.202
Emotional overeating	1.103	(0.658, 1.847)	0.710
Food avoidance traits			
Satiety responsiveness	0.529	(0.277, 1.013)	0.055
Slowness in eating	1.068	(0.671, 1.699)	0.782
Food fussiness	0.521	(0.327, 0.830)	**0.006**
Emotional undereating	0.848	(0.444, 1.623)	0.619

^1^Measured with the Child Eating Behavior Questionnaire.

^2^Adjusted for age at assessing appetitive traits, sex, gestational age, birthweight, paternal educational level, maternal educational level, household income, and sex-adjusted and age-adjusted body mass index standard deviation score at 5–6 years.

*p*-values < 0.05 are bolded.

### 3.4 Interactions between appetitive traits and biological sex

Supplementary Table 3 shows the interaction effects between appetitive traits and biological sex. No significant sex differences were observed in the associations between appetitive traits at 5–6 years and DDS 2 years later.

### 3.5 Inverse probability weighting

The pooled results from the weighted complete cases demonstrated that greater food fussiness at 5–6 years of age was associated with lower odds of high DDS 2 years later (OR = 0.332, 95% CI: 0.177–0.621), which was consistent with the main result. Furthermore, the pooled results indicated that a higher score for desire to drink (OR = 0.448, 95% CI: 0.248–0.809) was associated with a lower odd of high DDS. Greater satiety responsiveness and slowness in eating were also associated with lower likelihoods of high DDS (OR = 0.391, 95% CI: 0.211–0.724, and 0.529, 0.285–0.982, respectively). Meanwhile, greater enjoyment of food (OR = 2.011, 95% CI: 1.100–3.675) was associated with a higher likelihood of high DDS (Supplementary Table 4).

## 4 Discussion

As far as we know, this is the first longitudinal observational study to examine the associations between childhood appetitive traits (5–6 years of age, during kindergarten attendance) and dietary diversity 2 years later in primary school (7–8 years of age). We hypothesized that higher food approach traits in early childhood would be associated with a higher DDS, whereas higher food avoidance traits would be associated with a lower DDS. Consistent with our initial hypothesis, our study found that higher food fussiness, one of the key indicators reflecting a poor appetite, was significantly associated with decreased odds of achieving a high DDS. Meantime, our analyses did not identify any sex differences in the associations between appetitive traits and dietary diversity.

Food fussiness, or picky eating, is defined by selective eating patterns (19). This eating trait is frequently associated with various adverse nutritional outcomes, such as underweight, failure to meet dietary recommendations, and inadequate nutrition (20). Numerous studies have revealed that food fussiness has genetic and neural underpinnings. A twin cohort demonstrated that food fussiness was inherited and relatively consistent from toddlerhood through adolescence (21). Meanwhile, neuroimaging evidence suggested that adolescents with high food fussiness showed neural activation patterns in line with reduced approach toward fruits and vegetables (22). However, findings regarding food fussiness and dietary diversity were inconsistent. A cross-sectional study reported no associations between food fussiness and dietary diversity among children aged 1–3 years (23), which differed from our longitudinal findings. This discrepancy may be attributed to two key factors: the difference in study design and age-related variations in parental feeding practices and children’s developmental autonomy. During early childhood, parents often exert control over children’s food intake, which may mask the direct effect of food fussiness on dietary intake (24). As children mature, they gain greater food knowledge, feeding skills, and autonomy in food choices, leading food fussiness to exert a stronger influence on dietary diversity (25). Environmental and family factors play a pivotal role in developing food fussiness. Greater food fussiness is more common among children who are exposed to meal-time distractions, rewarded for finishing meals, allowed to eat freely, or occasionally dined with family (23). These findings highlight the influence of family meal environments on food fussiness and support targeted interventions to assist parents in cultivating children food fussiness (23).

Pooled results by IPW found that greater enjoyment of food was associated with higher DDS 2 years later. Enjoyment of food reflects positive emotional responses to eating. Higher enjoyment of food was found associated with a lower proportion of ultra-processed food intake relative to minimally processed foods intake, supporting healthier dietary patterns (26). IPW analysis also revealed that greater satiety responsiveness and slowness in eating were associated with lower DDS. Satiety responsiveness refers to the capacity to regulate intake based on internal fullness signals. Individuals with high satiety responsiveness tend to cease eating when full, whereas those with low satiety responsiveness may continue eating (27). Accumulating evidence has found that lower satiety responsiveness is associated with higher body weight in children (28). Meanwhile, environmental factors, such as sleep duration (29), were shown to shape satiety responsiveness, suggesting its importance in forming eating behaviors (30). Slowness in eating reflects eating rate. A two-cohorts longitudinal study confirmed that slower eating speed predicted lower risk of eating disorders (11). IPW analysis also found that higher desire to drink was negatively associated with higher DDS. One possible explanation was that children with higher desire to drink may prefer sugar-sweetened beverages over other foods, leading to a narrower range of food consumption (31, 32).

Food responsiveness refers to sensitivity and reactivity toward food-related stimuli, which was reported to be an early marker predicting eating disorder symptomatology (11). Emotional overeating is characterized by greater food intake triggered by negative emotions (33), whereas emotional undereating refers to reduced intake under distress or pleasure (34). Evidence showed that emotional overeating and undereating were associated with BMI (35). In our study, no significant associations were found between food responsiveness, emotional overeating, emotional undereating and DDS.

Various dietary guidelines recommend consuming diverse foods in order to ensure adequate energy and micronutrient intake, which may reduce inflammation, lower the risk of mortality, and make numerous other health benefits (36). Therefore, dietary diversity is widely recognized as a good indicator of diet quality in children (37). Our findings may provide novel insights for developing targeted interventions to improve dietary diversity in school-age children, especially by focusing on appetitive traits such as food fussiness. Although appetitive traits are moderately heritable (38), they can be modified, and environmental factors make a substantial contribution (39). Particularly, parenting practices and family meal environments are crucial factors in reshaping children’s appetitive traits (23, 40). Parents should learn to respond appropriately to children’s hunger, avoid interrupting children during mealtimes, and frequently have meals with children (23, 41). Our study found that early childhood appetitive traits were associated with dietary diversity in school-age children. Therefore, it is meaningful to focus on appetitive traits to improve nutritional status.

The major strengths of our study were the facts that we tracked participants for 2 years and performed a complete case analysis with IPW to reduce selection bias. The weighted complete case analysis supported the association between food fussiness and DDS, and uncovered additional associations between other appetitive traits and DDS. There were also limitations to this study. Firstly, we only tracked children until they entered primary school. Future studies need to follow participants into adolescence and even adulthood. Secondly, the additional associations that weighted complete case analysis discovered imply the need for larger cohort studies in the future research. Thirdly, several crucial confounders, including family meal environments, feeding and parenting practices, were not assessed in this study (13, 23, 40), which might influence appetitive traits and dietary diversity. Future studies should take these factors into consideration. Finally, owing to limitations in follow-up duration, sample size, and study design, the interaction effects between appetitive traits and biological sex may be underestimated.

## 5 Conclusion

This longitudinal study demonstrated that early childhood appetitive traits, especially food fussiness, were associated with dietary diversity in school-age children. Greater food fussiness at 5–6 years of age was associated with decreased odds of achieving a high DDS after 2 years. Although appetitive traits are heritable, they can be modified through environmental influences, such as parenting practices and family meal environments. Our findings reveal the associations between early childhood appetitive traits and dietary diversity, and provide novel insights for promoting nutrition in children.

## Data Availability

The raw data supporting the conclusions of this article will be made available by the authors, without undue reservation.
