# Supplementary material for: Associations between early childhood appetitive traits and dietary diversity in school-age children: a two-year longitudinal follow-up study
Source: Front Nutr. 2026 Jul 21;13:1824771. doi: 10.3389/fnut.2026.1824771 (PMC13433194; doi:10.3389/fnut.2026.1824771)
Supplement: Supplementary file 1 [file Table_1.docx]

Supplementary Material

**STable 1.** Characteristics of the final study sample compared to those lost to follow-up for dietary diversity score two years later

| Characteristics | Final study sample  (n = 194)  n (%) or mean ± SD | Non-response sample ^1^  (n = 14)  n (%) or mean ± SD | *p*-value ^2^ |
| --- | --- | --- | --- |
| Age at assessing appetitive traits, years | 5.59 ± 0.33 | 5.45 ± 0.34 | 0.131 |
| Sex |  |  | 0.483 |
| Female | 106 (54.64%) | 9 (64.29%) |  |
| Male | 88 (45.36%) | 5 (35.71%) |  |
| Gestational age, weeks | 39.06 ± 1.68 | 39.07 ± 1.38 | 0.975 |
| Birthweight, g | 3381.42 ± 569.53 | 3360.71 ± 481.66 | 0.895 |
| Sex-adjusted and age-adjusted BMI SD score at 5–6 years | 0.00 ± 1.52 | -0.16 ± 1.95 | 0.703 |
| Paternal educational level ^3^ |  |  | 0.908 |
| High | 125 (64.43%) | 9 (64.29%) |  |
| Medium | 60 (30.93%) | 4 (28.57%) |  |
| Low | 9 (4.64%) | 1 (7.14%) |  |
| Maternal educational level ^4^ |  |  | 0.299 |
| High | 125 (64.43%) | 11 (78.57%) |  |
| Medium | 63 (32.47%) | 2 (14.29%) |  |
| Low | 6 (3.09%) | 1 (7.14%) |  |
| Household income ^5^ |  |  | 0.740 |
| ＜150,000 yuan | 61 (31.44%) | 5 (35.71%) |  |
| ≥ 150,000 yuan | 133 (68.56%) | 9 (64.29%) |  |

^1^ The non-response sample was defined as participants with information available on appetitive traits in 5–6 years old but were lost to follow-up and did not have information available on dietary diversity score two years later (outcome).

^2^ *p*-values reflect differences by characteristic between the final study sample and the non-response sample and were obtained by t-tests or chi-square tests.

^3^ Paternal educational level was measured when assessing children’s appetitive traits (high: college or above, medium: vocational training, low: high school or below).

^4^ Maternal educational level was measured when assessing children’s appetitive traits (high: college or above, medium: vocational training, low: high school or below).

^5^ Household income was measured when assessing children’s appetitive traits.

BMI SD: body mass index standard deviation.

**STable 2.** Appetitive traits of the final study sample compared to those lost to follow-up for dietary diversity score two years later ^1^

| Appetitive traits | Final study sample  (n = 194)  mean ± SD | Non-response sample ^2^ (n = 14)  mean ± SD | | *p*-value ^3^ | |
| --- | --- | --- | --- | --- | --- |
| **Food approach traits** | | | | |  |
| Food responsiveness | 2.24 ± 0.92 | 1.80 ± 0.73 | | 0.084 | |
| Enjoyment of food | 3.32 ± 0.95 | 3.07 ± 0.94 | | 0.347 | |
| Desire to drink | 2.25 ± 0.99 | 1.64 ± 0.79 | | **0.026** | |
| Emotional overeating | 1.95 ± 0.75 | 1.30 ± 0.64 | | **0.002** | |
| **Food avoidance traits** | | |  |  |  |
| Satiety responsiveness | 2.64 ± 0.78 | 2.86 ± 0.87 | | 0.316 | |
| Slowness in eating | 2.84 ± 0.94 | 2.80 ± 0.67 | | 0.902 | |
| Food fussiness | 2.82 ± 0.82 | 2.79 ± 0.68 | | 0.887 | |
| Emotional undereating | 2.57 ± 0.56 | 2.07 ± 0.56 | | **0.002** | |

^1^ Measured with the Child Eating Behavior Questionnaire.

^2^ The non-response sample was defined as participants with information available on appetitive traits in 5–6 years old but were lost to follow-up and did not have information available on dietary diversity score two years later (outcome).

^3^ *p*-values reflect differences by appetitive traits between the final study sample and the non-response sample and were obtained by t-tests. *p*-values < 0.05 are bolded.

**STable 3.** Interactions between appetite traits at 5–6 years and biological sex on dietary diversity scores at 7–8 years ^1^

| Appetitive traits by sex | Dietary diversity score | | |
| --- | --- | --- | --- |
|  | OR | 95% CI | *p*-value |
| **Food approach traits** | | | |
| Food responsiveness by sex | 0.765 | (0.298, 1.962) | 0.577 |
| Enjoyment of food by sex | 0.822 | (0.296, 2.280) | 0.706 |
| Desire to drink by sex | 1.235 | (0.539, 2.828) | 0.618 |
| Emotional overeating by sex | 0.682 | (0.237, 1.965) | 0.478 |
| **Food avoidance traits** | | | |
| Satiety responsiveness by sex | 0.401 | (0.106, 1.509) | 0.177 |
| Slowness in eating by sex | 1.508 | (0.576, 3.951) | 0.403 |
| Food fussiness by sex | 0.903 | (0.350, 2.329) | 0.832 |
| Emotional undereating by sex | 1.588 | (0.405, 6.223) | 0.507 |

^1^ adjusted for age at assessing appetitive traits, sex, gestational age, birthweight, paternal educational level, maternal educational level, household income, and sex-adjusted and age-adjusted body mass index standard deviation score at 5–6 years.

**STable 4.** Inverse probability weighting adjusted binary logistic regression models showing the associations between appetitive traits at 5–6 years and dietary diversity scores at 7–8 years ^1^

| Appetitive traits | Dietary diversity score | | |
| --- | --- | --- | --- |
|  | OR | 95% CI | *p*-value |
| **Food approach traits** | | | |
| Food responsiveness | 0.717 | (0.394, 1.306) | 0.277 |
| Enjoyment of food | 2.011 | (1.100, 3.675) | **0.023** |
| Desire to drink | 0.448 | (0.248, 0.809) | **0.008** |
| Emotional overeating | 0.741 | (0.399, 1.375) | 0.342 |
| **Food avoidance traits** | | | |
| Satiety responsiveness | 0.391 | (0.211, 0.724) | **0.003** |
| Slowness in eating | 0.529 | (0.285, 0.982) | **0.044** |
| Food fussiness | 0.332 | (0.177, 0.621) | **0.001** |
| Emotional undereating | 0.552 | (0.305, 1.002) | 0.051 |

^1^ adjusted for age at assessing appetitive traits, sex, gestational age, birthweight, paternal educational level, maternal educational level, household income, and sex-adjusted and age-adjusted body mass index standard deviation score at 5–6 years.

*p*-values < 0.05 are bolded.
